# Two novel genetic variants in the mineralocorticoid receptor gene associated with spontaneous preterm birth

**DOI:** 10.1186/s12881-015-0205-y

**Published:** 2015-08-11

**Authors:** Inge Christiaens, Q. Wei Ang, Lindsay N. Gordon, Xin Fang, Scott M. Williams, Craig E. Pennell, David M. Olson

**Affiliations:** Obstetrics and Gynaecology, Newcastle University, Newcastle upon Tyne, NE2 4HH UK; School of Women’s and Infants’ Health, The University of Western Australia, Perth, 6009 WA Australia; Department of Genetics, Dartmouth College, Hanover, NH 03755 USA; Departments of Obstetrics and Gynecology and Pediatrics, University of Alberta, Edmonton, Alberta T6G 2S2 Canada; Center for Human Genetics Research, Vanderbilt University, Nashville, TN 37232-0700 USA

**Keywords:** Preterm birth, Chronic maternal stress, Single nucleotide polymorphism, Mineralocorticoid receptor

## Abstract

**Background:**

Preterm birth is the leading cause of mortality and morbidity in newborn infants. Its etiology is multifactorial with genes and environmental factors, including chronic maternal stress, contributing to its risk. Our objective was to investigate whether single nucleotide polymorphisms (SNPs) in genes involved in the stress response are associated with spontaneous preterm birth using a candidate gene approach.

**Methods:**

A total of 210 cases (singleton spontaneous preterm birth at <37 weeks) and 412 controls (singleton term birth at 38–42 weeks without a history of preterm birth) were studied. High quality maternal DNA was available from saliva samples of 190 cases and 369 controls and compared. Sociodemographic and medical data were collected. Sixteen SNPs, either tag SNPs located in key genes involved in the stress response identified in the Preterm Birth Genome Project database or SNPs found to be associated with adverse mental health outcomes in the published literature, were selected for genotyping and sequencing. SNPs were genotyped using Taqman® SNP genotyping assays. Univariate and multivariate logistic regression were performed.

**Results:**

Multivariate analysis showed that two SNPs located in the mineralocorticoid receptor gene were significantly associated with spontaneous preterm birth: rs17484063 (OR 0.50, p = 0.038) and rs2883929 (OR 0.49, p = 0.017), regardless of maternal age, smoking, alcohol use, educational status, and history of spontaneous miscarriage.

**Conclusion:**

This report demonstrates an association between mineralocorticoid receptor gene polymorphisms, rs17484063 and rs2883929, and preterm birth, supporting a role for genetics in the association between chronic maternal stress and preterm birth. Potentially, this information may be used to predicting the risk of having a preterm delivery.

## Background

Preterm birth (<37 weeks of gestation) is the leading cause of mortality in newborn infants and can lead to significant neonatal morbidities [1, 2]. Genetic predisposition plays an important role in the etiology of preterm birth; twin studies estimate the heritability of preterm birth to be 20 to 40 percent [3–9], and women born preterm have an increased risk of delivering their infants preterm. At present, the best predictor for preterm birth in multiparous women is a previous preterm birth, with the recurrence risk increasing with each additional preterm delivery. Preterm birth rates also significantly differ among ethnic groups with the largest racial disparity existing between non-Hispanic whites and women of African descent, even when corrected for lifestyle factors and demographic risk factors, such as age, socio-economic status and proper access to health care [10, 5].

Single nucleotide polymorphisms (SNPs) are the most common genetic variations accounting for approximately 90 percent of all human genetic variation; there is approximately one SNP per 300 base pairs [11]. SNPs can influence a person’s susceptibility to diseases or health outcomes such as preterm birth and can serve as markers of other types of variation, for example copy number variants. The relationship between SNPs and preterm birth has been extensively studied over the past decade; a synopsis of all genetic association studies that are published in the field of preterm birth can be found in the online PTBGene database [http://ric.einstein.yu.edu/ptbgene/index.html] [12] which is associated with the Preterm Birth International Consortium’s (PREBIC) Preterm Birth Genome Project (PGP) [http://www.prebic.org/projects-and-documents.html]. To date, more than 1600 SNPs in 80 candidate genes in putative preterm birth pathways have been analyzed in both maternal and fetal DNA samples; however, of all these SNPs, only three variants were found to be significantly associated with preterm birth in meta-analyses, namely rs1042713 in the adrenergic beta-2 receptor gene (ADRB2), rs1799963 (coagulation factor II precursor; F2), and rs2430561 (interferon gamma; IFNG).

This is unfortunate as the candidate SNP approach provides a reasonable method to associate genetic variations with preterm birth risk and to elucidate potential causal mechanisms, especially when combined with precise phenotyping and a suitable strategy for selection of both candidate SNPs and subjects. We therefore considered that perhaps a more directed approach to selecting candidate SNPs focused upon those associated with chronic stress would provide more insight. We had just determined that overall chronic stress and especially adverse childhood experiences are associated with preterm delivery [13], and in an animal model that transgenerational stress is associated with preterm delivery and changes in behaviour [14]. Further, maternal stress is also associated with common mental health conditions including major depressive disorders and anxiety disorders [15] - conditions with heritability estimates of 40 to 50 percent [16].

To date, there have been few reports demonstrating an association between SNPs in stress response genes and preterm birth [17]. This study explores the relationship between spontaneous preterm birth and SNPs in genes regulating stress responses or mood. The specific aims of this study are to explore the relationship between spontaneous preterm birth and candidate SNPs (derived from published literature) associated with adverse mental health outcomes, major depressive disorder and anxiety disorders, and to identify tag SNPs located in genes involved in the hypothalamic-pituitary-adrenal (HPA) axis and meso-corticolimbic system and evaluate their relationship with spontaneous preterm birth.

## Methods

### Study participants

This study was part of a case–control study based in Edmonton, Canada, and described in our recent publication [13]. Cases were defined as mothers who gave birth to a singleton at less than 37 weeks gestation (preterm) as a result of spontaneous idiopathic preterm labour with intact membranes. Controls were mothers with either a spontaneous, uncomplicated birth of a singleton at 38 to 41 weeks of gestation or an (elective) uncomplicated caesarean section between 38 and 41 gestational weeks. Similarly, women with a history of preterm delivery in the control group were excluded. Other exclusion criteria included pre-eclampsia, placental abruption, uterine malformations, minor and major fetal malformations, HIV or AIDS, influenza, H1N1 and non-English speakers.

The Human Research Ethical Boards of the University of Alberta, Alberta Health Services and Covenant Health approved the study. Written informed consent was obtained from all subjects before participating in the study.

### Collection of demographic data

Demographic and medical data were primarily collected from medical charts and described in our publication [13]. It was not possible to obtain all desired data as medical records were often incomplete or certain demographic data was not recorded. Missing data were collected via self-report at enrolment and/or during follow-up telephone interviews. Ethnicity was reported by self-identification back three generations from both the maternal and paternal side, where possible.

### DNA collection and extraction

At enrolment, saliva was collected using the Oragene DNA Self-Collection kit (OG-500, DNA Genotek). This method is equivalent to blood regarding the yield and quality of DNA for downstream applications [18]. It is easy, non-invasive, stable and cost effective. After collection, DNA samples were stored at room temperature for up to two years until the time of extraction. DNA purification and extraction were performed manually using the manufacturer’s protocol. The DNA concentration of each sample was determined using the NanoDrop ND-1000 spectrophotometer (NanoDrop Technologies).

One set of aliquots was normalized to 50 ng/μL to a volume of 170 μL and shipped to the University of Western Australia in Perth, Australia for genotyping. Samples below the necessary threshold of 50 ng/μL were excluded from genotyping. A total of 190 case samples and 369 control samples were included.

### Selection of candidate genes

A total of 16 SNPs were selected for genotyping. The complete list of selected SNPs, including information regarding the associated gene and allele, is described in Table 1.

**Table 1:** List of selected polymorphisms for genotyping

SNP	Gene name	Inclusion based on	Allele	Genotype technology
rs9470080	*FKBP5*	Literature	C/T	Taqman
rs10482605	*NR3C1*	Literature/TagSNP PGP database	C/T	Taqman
Proxy: rs4128428				
rs6190	*NR3C1*	Literature	T/C	Failed to design
rs2070951	*NR3C2*	Literature	G/C	Taqman
rs5522	*NR3C2*	Literature	T/C	Taqman
rs4680	*COMT*	Literature	A/G	Taqman
rs110402	*CRHR1*	Literature	G/A	Taqman
rs6323	*MAOA*	Literature	G/T	Failed to design
rs852978	*NR3C1*	TagSNP	C/T	Sequenom
		PGP database		
rs2963155	*NR3C1*	TagSNP	A/G	Sequenom
		PGP database		
rs17484063	*NR3C2*	TagSNP	C/T	Sequenom
		PGP database		
rs2883929	*NR3C2*	TagSNP	A/G	Sequenom
		PGP database		
rs4835136	*NR3C2*	TagSNP	C/T	Sequenom
		PGP database		
rs6826213	*NR3C2*	TagSNP	C/T	Sequenom
Proxy: rs7680420				
		PGP database		
rs173365	*CRHR1*	TagSNP	G/A	Sequenom
		PGP database		
rs1912151	*CRHR1*	TagSNP	T/C	Sequenom
		PGP database		

**Fig. 1:**
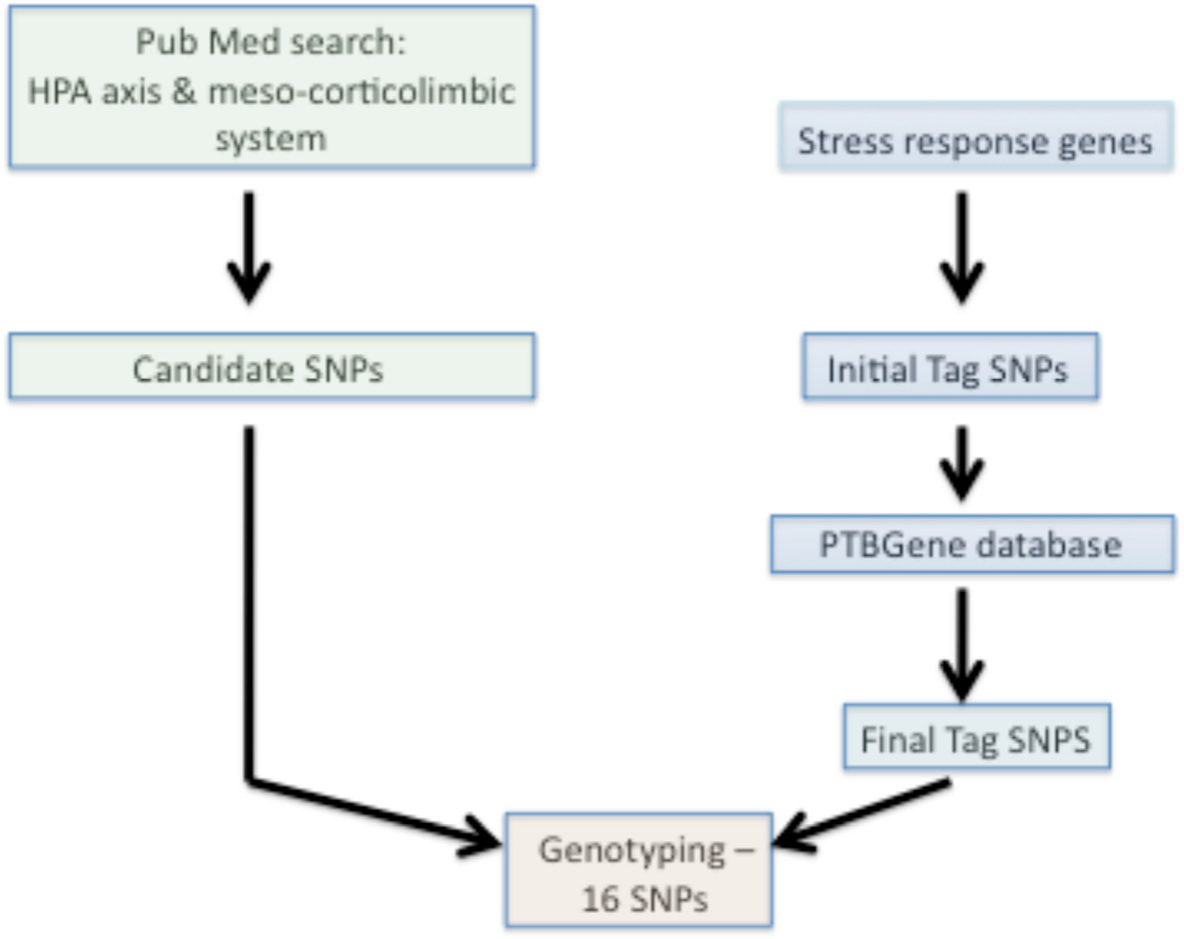
Selection of SNPs. For details, see Methods

Single nucleotide polymorphisms in genes involved in the stress response or mental health were selected using two different strategies (Fig. 1). To address the first part of our aim, a PubMed literature search was conducted to find published reports regarding genetic variants in genes involved in the regulation of the HPA axis and the meso-corticolimbic system and their associations with adverse mental health outcomes, especially major depressive disorder. Candidate SNPs were then selected based on prior associations with mental health outcomes as determined by a literature search. SNPs identified were added to our list of candidate SNPs. Additional SNPs were selected from seven key genes that are involved in the stress response and mood regulation. These genes code for the corticotropin releasing hormone receptor (CRHR1), the glucocorticoid receptor (NR3C1), the mineralocorticoid receptor (NR3C2), FK506 binding protein 5 (FKBP5), which is part of a receptor complex regulating the sensitivity of the glucocorticoid receptor, the serotonin transporter (SLC6A4), and the enzymes monoamine oxidase A (MAOA) and catechol-O-methyl transferase (COMT) that control the metabolism of serotonin, dopamine and norepinephrine in the brain. Tag SNPs for these seven genes were identified using the TagSNP function on the SNPinfo web server and the CEU samples from HapMap [19, 20]. Tagging conditions included an r^2^ threshold of 0.8 and a minor allele frequency (MAF) range of 0.05 to 0.5. This approach provided a broad coverage of functional (derived primarily from the literature) and non-functional SNPs and address most common genetic variations within these genes. All identified tag SNPs were then compared to the PGP database, and SNPs that were found to be associated with preterm birth in this database at *p* < 0.05 were selected for the candidate gene study. As a result, nine tag SNPs in three different genes – NR3C1, NR3C2 and CRHR1 – were included (Table 1).

### Genotyping

Genotyping was performed by the PathWest Molecular Genetics Service in Perth, Australia. SNPs were genotyped as per the manufacturer’s protocol using *Taqman*® SNP genotyping assays, designed and supplied by Applied Biosystems (ABI proprietary sequences). Perfect proxies based on the CEU samples from HapMap [21, 22] were used for two SNPs: rs7680420 for rs6826213 (NR3C2; r^2^ = 1.00), and rs4128428 for rs10482605 (NR3C1; r^2^ = 0.95). The SNPs rs6190 (NR3C1) and rs6323 (MAOA) were excluded from further analyses as no *Taqman*® probe could be designed.

In short, DNA samples were pipetted into 384-well PCR plates using a PerkinElmer Janus™ robot (4 ng DNA per well). The plates were allowed to dry at room temperature prior to use. Taqman genotyping reactions were carried out in a total volume of 3ul, containing 4 ng of dried template DNA, 1x *Taqman*® SNP genotyping assay, 200 μM dNTP (Promega), 3.5 mM MgCl_2_ (Applied Biosystems), 1X PCR buffer II (Applied Biosystems) and 0.18U AmpliTaq Gold® DNA Polymerase (Applied Biosystems). The reaction plates were incubated on a PCR thermal cycler for 10 min at 95 °C, followed by 40 cycles of 15 s at 92 °C and 1 min at 60 °C. Following PCR amplification, an allelic discrimination plate read was performed using an Applied Biosystems 7900HT Fast System. For all SNPs with a minor allele frequency greater or equal to 40 percent, samples of each genotype were sequenced to ensure that genotypes were called correctly. Samples of each genotype were used as controls in each experiment. To ensure that the data were reproducible, approximately 10 percent of the samples were genotyped in duplicate.

### Statistical analysis

All data were analyzed using SPSS 19.0 statistical software or STATA version 12. Before analyses, data sets were cleaned, and data from different sources were merged. Data were coded or recoded for analysis when required and missing data were indicated. Demographic and medical variables were compared between case and control subjects. For univariate analyses, variables were compared using logistic regression, and odds ratios (OR) and 95 percent confidence intervals (95 % CI) were recorded. Both education and miscarriage variables were dichotomized as high school or below vs. above high school and no prior miscarriage vs. any number of prior miscarriages in women who were not nulliparous. A *p*-value < 0.05 was considered significant. All SNPs were tested for Hardy-Weinberg equilibrium (HWE) by Chi-squared test, using PLINK [23].

To assess the relationship between each SNP and spontaneous preterm birth, univariate logistic regression was performed using an additive model, except rs5522, rs852978, and rs7680420, which used a dominant model by collapsing the rare homozygote of the minor allele with the heterozygotes. Odds ratios, 95 % CIs and p-values were calculated. A p-value < 0.05 was considered significant. Next, a multivariate model was created including those demographic variables that were significantly different between cases and controls in our population, including maternal age, smoking, alcohol, education status, and previous miscarriage. Adjusted ORs and 95 % CIs were calculated with the multivariate logistic model. P-values < 0.05 were considered significant. Results were not adjusted for multiple tests.

When multiple SNPs within one gene associated with risk of preterm birth, haplotypes consisting of all the SNPs within a gene were tested to determine if these SNPs exert their effects independently of each other. To visualize linkage disequilibrium (LD) structure within a given gene, Haploview [24] generated LD plots depicting r^2^ values in cases and controls separately. The effect of haplotypes within a gene was calculated in UNPHASED, where 2-, 3-, and 4- SNP haplotypes were tested for association with preterm birth [25]. To determine significance and effect size of the haplotype of interest, this haplotype was compared against all other haplotypes to estimate the OR, 95 % CI, and p-value. Additionally, the global, or omnibus, test indicates whether any of the haplotypes are associated. The omnibus test compares all haplotypes to the most common one. For each possible combination of SNPs consisting of a haplotype, only the most significant haplotype was reported. Significance in UNPHASED is derived from a permutation test which corrects for multiple tests.

## Results

A total of 622 women (210 cases, 412 controls) were initially included in the large case–control study that examined both genetic variants and environmental factors including chronic stress (see reference 13). Numbers among tests depend on the availability of either genotypes or demographic data.

### Univariate analyses

All socio-demographic and medical variables were compared and described between the case and control group and their possible relationship with spontaneous preterm birth was assessed (Table 1 in reference 13).

For the candidate gene studies, a total of 190 case samples and 369 control samples were analyzed. No SNPs showed a deviation from Hardy Weinberg Equilibrium with a p < 0.01 (data not shown). Therefore, all SNPs were included in subsequent analyses.

Univariate analysis showed that four tag SNPs, all located in the mineralocorticoid receptor gene, NR3C2, were associated with spontaneous preterm birth in our study (Table 2). These SNPs were rs17484063, rs2883929, rs4835136, and rs7680420 (crude ORs 0.71, 0.63, 0.66, and 1.63 respectively). None of the other selected tag SNPs and candidate SNPs based on the literature showed a significant association with preterm birth.

**Table 2:** Univariate analysis of candidate SNPs selected based on literature and TagSNPs in genes involved in mental health pathways

SNP	Gene	Crude OR^a^	*p*-value
rs4128428	NR3C1	1.18	0.35
rs2070951	NR3C2	1.09	0.52
rs5522	NR3C2	1.33	0.14
rs4680	COMT	1.01	0.95
rs852978	NR3C1	0.70	0.08
rs2963155	NR3C1	0.75	0.055
rs17484063	NR3C2	0.71	0.047
rs2883929	NR3C2	0.63	0.005
rs4835136	NR3C2	0.66	0.004
rs7680420 (proxy)	NR3C2	1.60	0.017
rs173365	CRHR1	0.94	0.61
rs1912151	CRHR1	0.91	0.56

Variables were analyzed using univariate logistic regression (additive model)

^a^Referent for OR is the major allele at each site

### Multivariate analysis

Univariate analyses of the socio-demographic and medical variables demonstrated that maternal age, smoking, alcohol and drug use, educational status, and a history of miscarriage were significantly associated with preterm birth in our study population. In our multivariate model we therefore included maternal age, smoking, alcohol use, education, and history of miscarriage as covariates. We excluded drug use since the number of respondents reporting drug use was very small (n < 5).

The NR3C2 polymorphisms rs17484063, rs2883929, rs4835136, and rs7680420 were all analyzed utilizing the multivariate model. After adjustment, two SNPs, rs1784063 and rs2883929, remained significantly associated with decreased spontaneous preterm birth risk (OR 0.50; 95 % CI 0.26-0.96 and OR 0.49; 95 % CI 0.27-0.88. The results are summarized in Table 3.

**Table 3:** Multivariate analysis of four SNPs located in the mineralocorticoid receptor gene

SNP	Adjusted OR^a^	95 % CI	*p*-value
rs17484063	0.50	0.26-0.96	0.038
rs2883929	0.49	0.27-0.88	0.017
rs4835136	0.62	0.38-1.02	0.060
rs7680420	1.73	0.90-3.31	0.099

^a^Referent for OR is the major allele at each site

For rs1784063 and rs2883929 the minor alleles were protective or decreased risk (Adjusted OR = 0.50 and 0.49, respectively). In contrast, rs4835136 and rs7680420 were not significantly associated with spontaneous preterm birth after adjustment (OR 0.62; 95 % CI 0.38-1.02 and OR 1.73; 95 % CI 0.90-3.31).

**Fig. 2:**
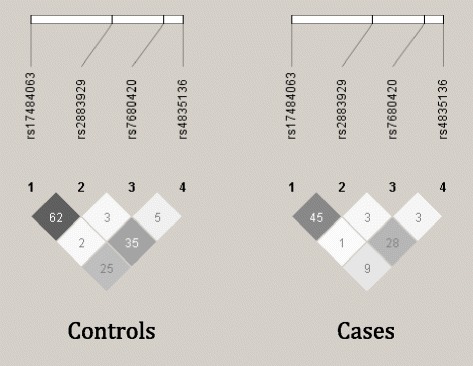
The two associated SNPs, rs17484063 and rs2883929, are in stronger LD, as measured by r^2^, in the controls than the cases (p = 0.007). LD patterns in *NR3C2* differ between cases and controls

**Fig. 3:**
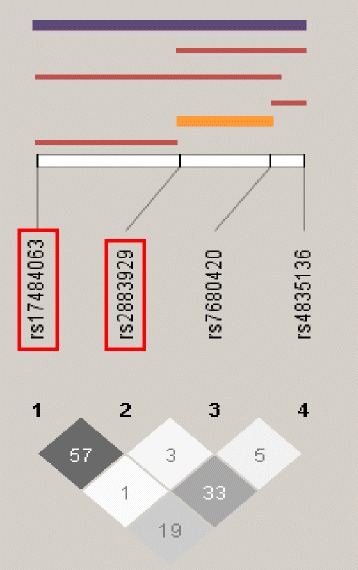
Haplotypes associating with the greatest p-value for combinations of all possible linear 2-, 3-, 4-SNP haplotype windows are shown. The haplotype with the most significant association in the global test is shown in orange, while the haplotype with the most significant association against all other haplotypes is in purple. The two SNPs boxed in red associated with decreased risk of preterm birth in the multivariate model. *NR3C2* haplotypes associate with decreased risk of preterm birth

Since the four most significant SNPs by univariate analyses were located within *NR3C2*, we tested whether these SNPs influence risk independently of each other. When adjusting for the effects of all other *NR3C2* SNPs, none of the *NR3C2* SNPs associate with preterm birth risk and indeed they were protective (Table 3). LD structure among these four SNPs was also examined in cases and controls separately, which showed r^2^ values between both the associated SNPs, rs17484063 and rs2883929, but also the other *NR3C2* SNPs is higher in the controls than the preterm birth cases (Fig. 2; p = 0.007 by t-test). Additionally, the different haplotypes created by these SNPs were tested for association with preterm birth. Haplotypes consisting of 2-, 3-, and 4-SNP windows all associated with preterm birth (Fig. 3 and Table 4). Additionally, the 4-SNP haplotype, T-G-T-T, was the most significantly associated haplotype when tested against all other 4-SNP haplotypes (OR 0.41; 95 % CI 0.23-0.73) while the 2-SNP haplotype, consisting of the last two SNPs in *NR3C2*, T-T, was the most significantly associated 2-SNP haplotype by the global test (OR 0.51; 95 % CI 0.33-0.79).

**Table 4:** Association of *NR3C2* haplotypes with decreased risk of preterm birth. Continuous haplotype with the most significant association presented here for haplotypes consisting of two, three, and four *NR3C2* alleles

*NR3C2* SNPs	Reference haplotype	Associated haplotype	Case frequency	Control frequency	Odds ratio	95 % CI	*p*-value
1-2	C-A	T-G	0.11	0.18	0.58	0.40-0.85	0.0048
2-3	A-T	G-T	0.16	0.24	0.66	0.48-0.92	0.0043
3-4	T-C	T-T	0.26	0.36	0.51	0.33-0.79	0.0020
1-2-3	C-A-T	T-G-T	0.12	0.18	0.45	0.27-0.76	0.0054
2-3-4	A-T-C	G-T-T	0.13	0.21	0.44	0.27-0.73	0.0027
ALL	C-A-T-C	T-G-T-T	0.09	0.16	0.41	0.23-0.73	0.0011

Associated haplotypes were compared against all other haplotypes to determine significance. *NR3C2* SNPs are enumerated 1–4 with the numbers representing rs17484063, rs2883929, rs4835136, and rs7680420, respectively

## Discussion

Our candidate gene studies have demonstrated that the minor alleles of polymorphisms rs17484063 and rs2883929, both located in NR3C2 gene coding for the mineralocorticoid receptor, are significantly associated with reduced risk for spontaneous preterm birth. This association was independent of the known preterm birth risk factors maternal age, smoking status, alcohol use, educational status and history of miscarriage. For women who are heterozygous for the rs17484063 or the rs2883929 effect allele, the risk of spontaneous preterm birth was halved. If a woman is homozygous for either risk allele, the risk of delivering preterm is further halved. This is the first report demonstrating the association between these polymorphisms and preterm birth. Haplotype analyses also revealed a ~35 % to 60 % reduction in risk relative to the referents.

While previous studies have examined SNPs related to the HPA axis [26, 27], no significant associations for either rs17484063 or rs2883929 have been published and therefore little is known about them. Rs17484063 is a noncoding SNP located on Chromosome 4 in the intron region of NR3C2. The alleles for rs17484063 are C/T, with T being the minor or effect allele. The MAF in our population was 0.18. The SNP function prediction tool on the SNPinfo web server indicates that rs17484063 is non-functional [20]. This was to be expected, as it is located in the intron region of NR3C2. Rs17484063 is a tag SNP representing a haplotype block of five SNPs all in strong LD with rs17484063 (r^2^ > 0.8): rs10519951, rs17484063, rs17484118, rs17484259, and rs17581262. Two SNPs, rs17581262 and rs17484118, are also in close LD with rs2883929. All SNPs in this haplotype block are located in the intron region of NR3C2. These results indicate that none of our genotyped SNPs are likely to be the functional ones with respect to PTB.

Rs2883929 is also a noncoding SNP located in the intron region of NR3C2. Its alleles are A and G, with G being the minor allele. The MAF in our population was 20 percent. Similar to rs17484063, rs2883929 is non-functional. It is a tag SNP representing a haplotype block of eight SNPs: rs11936376, rs17484118, rs17581262, rs17581570, rs2356210, rs2883929, rs3846326, and rs7689925. All SNPs of this haplotype block are in strong LD with rs2883929 (r^2^ > 0.8) and are located in the intron region of NR3C2. Two SNPs - rs17581262 and rs17484118 – were found to be in close LD with both tag SNPs rs17484063 and rs288392.

As the two SNPs associated with spontaneous preterm birth are both intronic, it is unclear what their functional significance might be, if there is any. When exploring the SNP function potential on the SNPinfo web server of all SNPs in the two haplotype blocks, the polymorphisms rs17581262 and rs3846326 showed a low regulatory potential score of 0.09 and 0.01, respectively. Sequencing of these regions in combination with functional studies may provide more insight into the biological roles of rs17484063 and rs2883929 and preterm birth.

Cortisol is the main effector of HPA axis and the stress response. Its actions are exerted when cortisol is bound to the glucocorticoid (GR) and mineralocorticoid (MR) receptors. While the GR is present in almost every cell in the body [28], the MR occurs mainly in brain areas of the limbic system and the hippocampus [29]. The affinity of cortisol for the MR is much greater than for GR, and binding of cortisol to MR is maintained at basal levels while the GR becomes activated in response to a stressor. It is thought that the MR mainly regulates the onset of activation of the HPA axis, while the GR regulates the termination of activity [30]. Functional polymorphisms in the human NR3C2 gene have been identified [29, 31, 32]. These were found to be associated with increased levels of cortisol and psychosocial stress, but to date, no reports on a possible link between polymorphisms in NR3C2 and preterm birth have been published. As genetic variants in the MR gene were found to upregulate the expression and activity of MR and are associated with higher levels of cortisol and perceived chronic stress, it is biologically plausible that SNPs in NR3C2 are associated with preterm birth.

## Conclusion

We demonstrated that the mineralocorticoid receptor gene polymorphisms rs17484063 and rs2883929 are significantly associated with spontaneous preterm birth, with the minor alleles associating with reduced risk. Although the functional roles of these two polymorphisms remain unclear, it was shown in our study that they might play a protective role in the etiology of spontaneous preterm birth.

## Abbreviations

SNP: Single nucleotide polymorphism
GWAS: Genome-wide association studies
PGP: Preterm Birth Genome Project
HPA: Hypothalamic-pituitary-adrenal
CRH: Corticotropin releasing hormone
ACTH: Adrenocorticotropic hormone
GR: Glucocorticoid receptor
BMI: Body mass index
PCR: Polymerase chain reaction
MAF: Minor allele frequency
OR: Odds ratio
CI: Confidence interval
HWE: Hardy-Weinberg equilibrium
LD: Linkage disequilibrium
MR: Mineralocorticoid receptor
